# Functional analysis of *CYP6ER1*, a P450 gene associated with imidacloprid resistance in *Nilaparvata lugens*

**DOI:** 10.1038/srep34992

**Published:** 2016-10-10

**Authors:** Rui Pang, Meng Chen, Zhikun Liang, Xiangzhao Yue, Hu Ge, Wenqing Zhang

**Affiliations:** 1 State Key Laboratory of Biocontrol, School of Life Sciences, Sun Yat-sen University, Guangzhou, 510006 China; 2 School of Pharmaceutical Science, Sun Yat-sen University, Guangzhou, 510006 China

## Abstract

The cytochrome P450 *CYP6ER1* has been reported to play an important role in imidacloprid resistance of the brown planthopper (BPH), *Nilaparvata lugens*, and is overexpressed in most resistant populations. In the present study, we confirmed that *CYP6ER1* expression can be induced by certain levels of imidacloprid. Developmental expression analysis revealed that *CYP6ER1* was expressed highly in the adult stage, and tissue distribution analysis showed that *CYP6ER1* was expressed mainly in the fat body and midgut. RNA interference (RNAi) of *CYP6ER1* and transgenic expression of *CYP6ER1* in *Drosophila melanogaster* both suggested that the expression of *CYP6ER1* is sufficient to confer imidacloprid resistance. Furthermore, we analyzed the interaction of imidacloprid and CYP6ER1 monooxygenase by using dynamic simulations and molecular docking. We found that Nitrogen atoms in the heterocycle of the imidacloprid molecule may bind to iron atoms in the center of the homology model of CYP6ER1 via 4,5-dihedro-1H-imidazole. This finding contributes to a better understanding of how CYP6ER1 takes part in the insecticide metabolism.

The brown planthopper (BPH), *Nilaparvata lugens* is one of the monophagous rice insect pests, whose outbreaks in can cause great losses in rice producing regions. To protect rice crops from serious damage by *N. lugens*, the widespread application of insecticide has been implemented by many farmers^1^. However, after over 30 years of insecticide use to control rice planthopper infestations in China and other areas of Asia, it has become apparent that the chronic application of any kind of insecticide can lead to the development of resistance, and this resistance has emerged as a major problem in *N. lugens* control^2^. Within field populations of *N.lugens* resistance to major insecticide classes, such as organochlorines^3^,^4^, organophosphates^5^,^6^, carbamates^3^, pyrethroids^7^, buprofezin^8^, neonicotinoids^9^, fipronil^10^, and pymetrozine^11^, has emerged.

Insecticide resistance involves a variety of mechanisms, such as reduction of penetration rate, enhancement of metabolic detoxification, accelerated excretion of insecticides from the organism and target site insensitivity. Enhancement of P450 monooxygenase enzymatic activity related to insecticides resistance, especially to neonicotinoid insecticides, is a typical case of enhancement of metabolic detoxification^12^. *In vivo* suppression or decrease in resistance by application of P450 inhibitors such as the synergist piperonyl butoxide is often used as a diagnostic for P450 involvement^13^. In many cases, the over-expression or up-regulation of P450, can increase pests’ ability to resist to insecticide. An example of this is, *CYP6G1* overexpression in *Drosophila* and the associated resistance to DDT^14^. *CYP6D1* causes resistance to pyrethroid in house flies^15^, and *CYP4E3* expression results in permethrin insecticide tolerance in *Drosophila*^16^. In BPH, a single P450 gene, *CYP6ER1*, is thought to play an important role in imidacloprid resistance^17^,^18^,^19^. The overexpression of *CYP6ER1* has been found in most resistant strains and the level of expression observed in the different BPH strains was significantly correlated with the resistance phenotype^19^. In addition, a previous study indicated *CYP6ER1* metabolized imidacloprid less efficiently compared to *CYP6AY1* but it could be upregulated by imidacloprid^20^.

However, it is impossible to predict catalytic activity of P450s based on sequence alignments in order to give deeper insight into their mechanisms of insecticides metabolism^21^. Protein modeling is a method to obtain structural data when experimental techniques fail. Many proteins are too large for NMR analysis and cannot be crystallized for X-ray diffraction^22^, resulting in a lack of elucidate crystal structures for P450s and their substrate complexes. In these cases, homology modeling gives an insight into understanding the complexity of insecticide resistance mediated by P450 genes^23^,^24^. By comparing homologous candidates, a number of candidates searched in the database can, to a certain extent, allow researchers to predict and visualize specific molecular structures and interactions, which are difficult to prove using traditional biological experiments. Jones utilized homology modeling based on a template of the X-ray structure of the phylogenetically related human CYP3A4, to simulate the interaction between *D. melanogaster* CYP6G1 and DDT and neonicotinoids, and indicated roles for CYP6G1, CYP12D1 and CYP6A2 in flies overexpressing P450 enzymes with broad substrate specificities in chemical metabolism^25^. Karunker found interactions of imidacloprid with the biotype Q variant of the CYP6CM1 enzyme might involve key amino acids such as Phe-130 and Phe-226 in the enzyme active site with the lowest energy, and they predicted a putative hydroxylation site^26^. Unfortunately, while the application of homology modeling is now widely spread in the medical sciences, it has been used in only a limited capacity for the determination of insecticide metabolism mechanisms in pest control.

ln this study, we determined, using RNAi and transgenic expression in *Drosophila*, that the expression of P450 *CYP6ER1* is sufficient to confer imidacloprid resistance. Additionally, we utilized molecular modeling in order to predict the catalytic site geometrics and identify the key residues responsible for imidacloprid binding with CYP6ER1. These experiments contribute to clarifying the enzyme responsible for metabolizing imidacloprid and causing the resistance phenotype.

## Results

### *CYP6ER1* expression patterns in response to insecticide exposure

**Figure 1:**
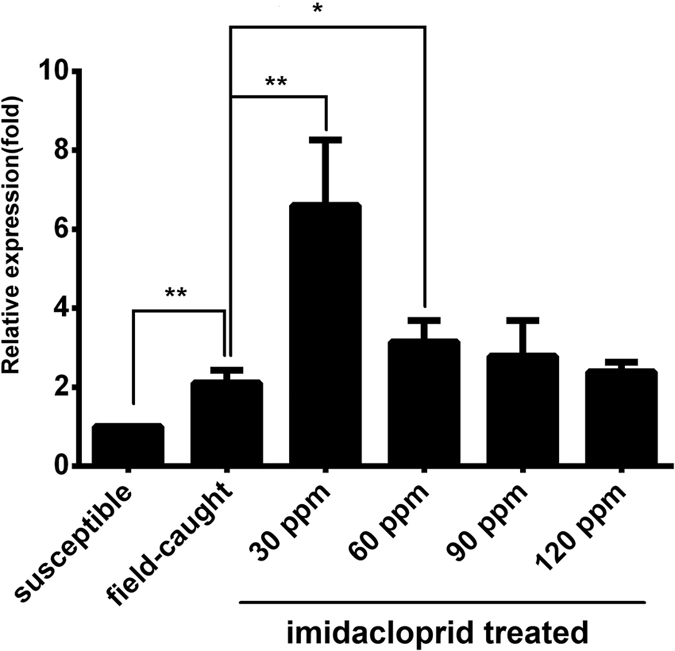
*CYP6ER1* expression patterns in response to insecticide exposure. Quantitative real-time PCR measurement of the fold change in the expression of *CYP6ER1* in the field-caught strain compared with the susceptible strain, while the field-caught strain treated with 4 different doses of imidacloprid (30ppm,60ppm,90ppm and 120ppm, respectively) compared with the field-caught strain without imidacloprid. *β*-actin was used as an internal reference gene and the mRNA expression level of *CYP6ER1* in the susceptible strains was designated as one. The data represent the mean + SEM (n = 30). *P < 0.05 level (*t*-test) and **P < 0.01 level (*t*-test).

In order to confirm the expression pattern of *CYP6ER1* in insecticide resistant BPH strains, one-day-old female adults of a field-caught strain from Guangxi and a susceptible contrasted strain from Zhejiang were selected and the field-caught strain was further treated with 4 different doses of imidachloprid. We found that the mRNA level of *CYP6ER1* in the field-caught strain was 2.1-fold higher than that observed for the susceptible strain, and the mRNA expression level in the samples treated with 30 ppm and 60 ppm imidachloprid was significantly higher than that observed for the untreated field-caught strain. However, there was no significant difference between the untreated field-caught strain and samples treated with 90 ppm or 120 ppm imidachloprid (Fig. 1). These results suggested that the up-regulation of *CYP6ER1* can be triggered by imidachloprid, and it can reach at maximum within certain dosages of imidachloprid.

### Developmental expression and tissue distribution of *CYP6ER1*

**Figure 2:**
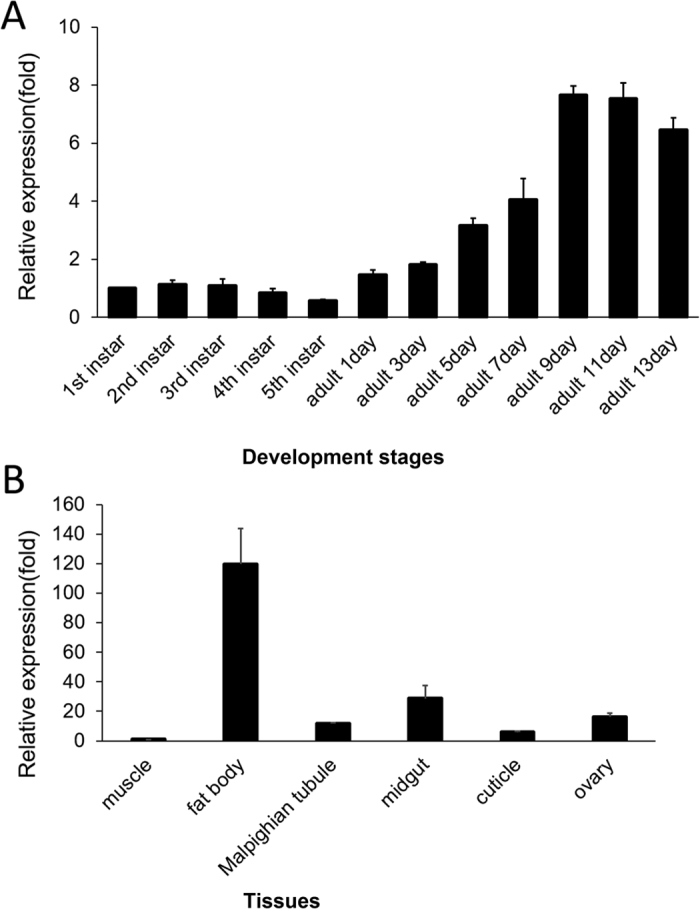
Developmental expression and tissue distribution of *CYP6ER1*. **(A)** Developmental expression of *CYP6ER1* mRNA by qRT-PCR.Nymphs1–5: the 1st instar nymphs to the 5th instar nymphs; Adults odd day of 1–13 days: female adults from the odd day of 1 to 13 days. The mRNA levels of *CYP6ER1* were relative to the *β-actin* mRNA level and the mRNA expression level in 1st instar are designated as one. The data represent the mean + SEM (n = 30). **(B)** Tissue distribution of *CYP6ER1*. Fat body, Ovary, Midgut, Cuticle, Malpighian tubule and muscle. The mRNA levels of *CYP6ER1* were relative to the *β*-actin mRNA level and the mRNA expression level in muscle is designated as one. The data represent the mean + SEM (n = 3).

Quantitative Real-time PCR (qRT-PCR) was used to detect the expression patterns of *CYP6ER1* during *N.lugens* from the first instar nymphs to the 13th day adulthood. The mRNA levels developed a stable trend of low expression before 5th instar, but increased significantly from adult day 1 to a relatively high level between adult day 9 and day 13 (Fig. 2A). We also sampled different tissues from 1-day-old female adults to investigate their mRNA levels of *CYP6ER1*, and it was expressed highly in the fat body and midgut, expressed at a relatively lower level in the ovaries, Malpighian tubule and cuticle, and not expressed in the muscle (Fig. 2B). The high levels of expression in the essential metabolic organs correspond to the physiological role of *CYP6ER1* in imidacloprid resistance.

### RNA interference of *CYP6ER1*

**Figure 3:**
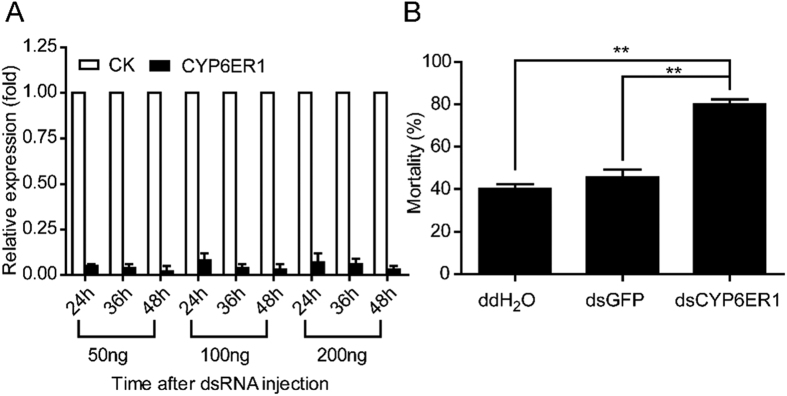
Detection of the mRNA level and mortality after RNA interference (RNAi). **(A)** The *CYP6ER1* mRNA expression level after injecting three doses (50 ng, 100 ng and 200 ng) of ds*CYP6ER1* at three time points. The mRNA expression level of CYP6ER1 in ds*GFP* ground and ds*CYP6ER1* group were relative to β-actin mRNA level and the mRNA expression level of *CYP6ER1* in ds*GFP* group was designated as one. **(B)** Effects on the mortality rate after injection of water or ds*GFP* or ds*CYP6ER1* and followed by application of imidacloprid (50 ng/ml). Double asterisks indicate significant differences in the mRNA relative expression level between the treatment and control groups (P < 0.01, t-test). The data represent the mean + SEM (n = 3), **P < 0.01 level (*t*-test).

To evaluate the contributions of *CYP6ER1* in imidacloprid resistance *in vivo*, we designed dsRNA to silence CYP6ER1 in female adult from the Guangxi strain. The mRNA expression of *CYP6ER1* decreased within 48 hours after injecting three doses (50 ng, 100 ng and 200 ng) of ds*CYP6ER1*, but there was no significant difference between the three doses of dsRNA (Fig. 3A). We chose 50 ng imidacloprid treatment for 48 hours to test BPH mortality after RNAi (Fig. 3B). The mortality of the ds*CYP6ER1* sample was 40.00% and 34.42% higher than those of the ddH_2_O and ds*GFP* samples, respectively. This suggests that the expression of *CYP6ER1* should be of great importance to imidacloprid resistance in BPH.

### Transgenic expression of the potential resistance genes *CYP6ER1* in *D. melanogaster*

**Figure 4:**
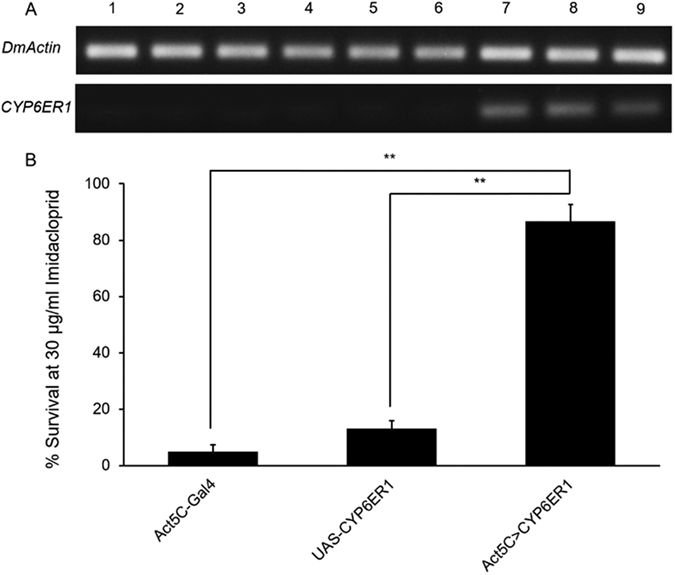
Transgenic expression of *CYP6ER1* in *D. melanogaster* and its effect on imidacloprid resistance. **(A)** The expressions of *CYP6ER1* were confirmed by RT-PCR in two control lines and transgenic line. 1, 2, 3, three biological replicates of Act5C-GAL4, flies with genetic background correspond to Act5C-GAL4; 4, 5, 6, three biological replicates of UAS-*CYP6ER1*, flies not expressing the *CYP6ER1*; 7, 8, 9, three biological replicates of Act5C>*CYP6ER1*, transgenic flies expressing the *CYP6ER1*. **(B)** The comparison between survival rates of two control lines and transgenic line exposed to 30 μg/ml imidacloprid. The data shown are the mean ± SEM (n = 3). **P < 0.01 (Chi-squared Test).

To identify whether the expression of *CYP6ER1* is sufficient to confer imidacloprid resistance, we used a transgenic approach utilizing the GAL4/UAS system of *D. melanogaster*. *CYP6ER1* was inserted into the vector pUAST and embryonic microinjections were performed on *D. melanogaster* w^1118^ to generate the transgenic germline carrying a UAS- *CYP6ER1* transgene. The flies subsequently were crossed to the Act5C-GAL4 line. The mRNA levels of *CYP6ER1* were determined in the progeny (1–3 days-old adults) by RT-PCR, confirming the expression of the transgene in the Act5C >*CYP6ER1* line (progeny expressing the *CYP6ER1*) (Fig. 4A). In comparison, the *CYP6ER1* mRNA was not detected in another two control *D. melanogaster* lines (UAS- *CYP6ER1* and Act5C-GAL4, progeny not expressing the *CYP6ER1*).

Bioassays showed that Act5C >*CYP6ER1* line was resistant to imidacloprid at a treated dose of 30 μg/ml imidacloprid, with significant higher survival rate (86.42%, *p* < 1e-10 Chi-squared Test) than the control lines (4.23% and 11.15% respectively) (Fig. 4B). These results suggest that the expression of *CYP6ER1*confers imidacloprid resistance.

### Homology modelling of CYP6ER1

**Figure 5:**
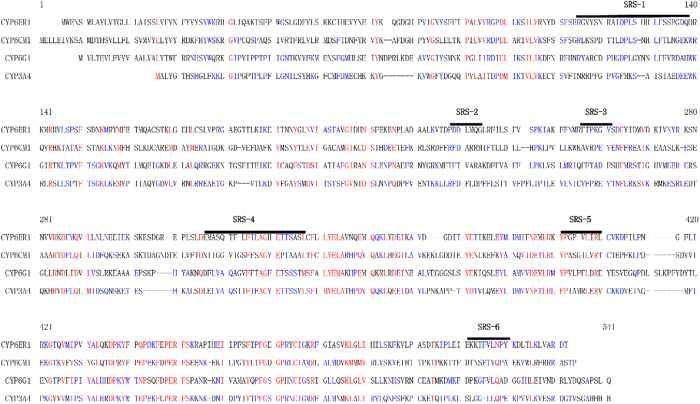
Sequence alignment of *CYP6CM1*vQ, *CYP3A4*, *CYP6ER1* and *CYP6G1*. Sequences are colored red for identity and colored blue for similarity. The substrate recognition site (SRS) regions are represented by a black line.

Structure based sequence alignment was used to compare the key residues previously predicted to be responsible for imidacloprid binding and metabolism, such as CYP6CMA1, CYP3A4, and CYP6G1 (Fig. 5; Table 1). CYP6ER1 had a high similarity and shared several functionally identical amino acids, suggesting that CYP6ER1 may have the same functional domain as other imidacloprid metabolism proteins. The prediction of the substrate recognition site (SRS) secondary structure elements and key residues were cited in the research of CYP6CM1^26^. A BLAST search with the default parameters for CYP6ER1 in the PDB_nr95 database identified the homologous protein, CYP3A4 (PDB code: 3NXU), with an intermediate sequence identity of 31%, which was then used to construct the CYP6ER1 structure.

A total of 20 models were generated. Among these 20 models, 6ER1.M0011 had the smallest value of PDF (probability density function) Total Energy and PDF Physical Energy, and relatively low DOPE Score (Table S3), by using Cut overhangs, refine loops and use of DOPE method as parameters. This indicated that 6ER1.M0011 was the most suitable model among the 20 counterparts, and could be utilized to simulate the molecular docking. 6ER1.M0011 was then analyzed by Ramachandran and most of these residues were located in the most favored region, with 11 out of 470 residues in the disallowed region, suggesting it was a relatively good quality model but needed further regulation and optimization.

### The binding mode of imidacloprid to the CYP6ER1 active site

During our analysis, we found ritonavir as specific inhibitor of CYP3A4 in human, which suggested that its docking method may be similar to CYP6ER1. Imidachloprid and ritonavir both have a similar heterocycle (Fig. S1A,B) and ESP charge distribution (Fig. S1C,D). Rritonavir bonds covalently to CYP3A4 via nitrogen atoms, and this mode may be similar to the way imidachloprid bonds to CYP6ER1.

**Figure 6:**
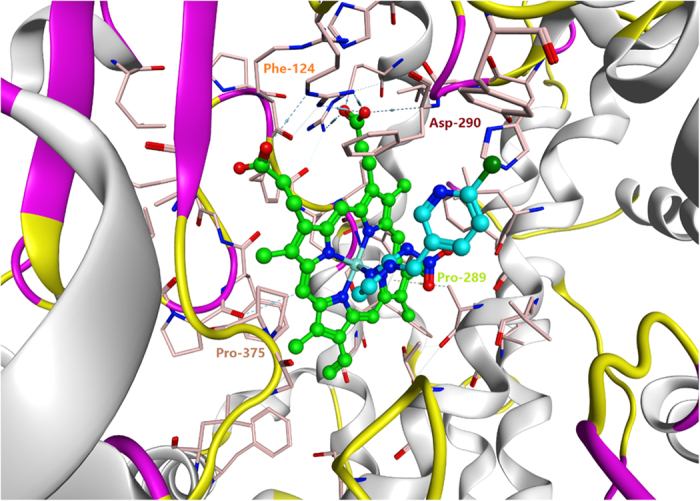
Overview of P450-HEME-IM4 complex and its binding pocket. The one conformation of MD-based optimized structure. Imidacloprid is indicated with light blue carbon atoms and the HEME is showed with grass green atoms. Predicted binding residues within 4 Å of imidacloprid are showed in pink. Phe-124 and Asp-290 anchor imidacloprid by aromatic interactions, and generate significant part of the contact surface area. Imidacloprid may be further stabilized by hydrogen bonded with Pro-375 and Pro-289.

We used the homology model based on CYP6ER1 as the receptor, and the imidachloprid molecule as the ligand. Nitrogen atoms in the heterocycle of the imidachloprid molecule bonded to iron atoms in the center of the homology model via 4,5-dihedro-1H-imidazole. The temperature of CYP6ER1-imidacloprid compound hovered around 300 K, with its deviation below 10 K (Fig. S2), and the total energy, kinetic energy and potential energy started from high energy to a stable relatively low level after about 5 picoseconds (Fig. S3). RMSD of atomic coordination of the protein backbone, HEME and ligand fluctuated up and down in the range between 1.20 Å to 1.50 Å (Fig. S4). The results confirmed the hypothesis of the formation of a CYP6ER1-imidacloprid complex based on its relatively credible stability and balance of its energy and the structure. Finally, the interaction energy of CYP6ER1-imidacloprid complex was calculated at −114.66 kcal/mol, indicating that this may be the ideal model to simulate the P450-HEME-IM4 complex and its binding pocket, and suggested that some amino acids in the center of CYP6ER1 have a role in the molecular docking the between CYP6ER1 and imidachloprid (Figs 5 and 6 and Table 1). Most of these residues were predicted to generate the hydrophobic interface of the binding cavity, in which Phe-124 was predicted to anchor imidacloprid by aromatic interaction generating the largest hydrophobic patch in the interface. We predicted that imidacloprid would be further stabilized by hydrogen donors and acceptors that include Pro-375 (SRS-5) and Pro-289 (SRS-2), which can interact with imidacloprid’s imine moiety and Leu-312(SRS-4).

## Discussion

It is generally believed that detoxifying enzymes, referred to as esterase, glutathione S-transferase and P450s, contribute to defending against and catabolizing toxins and insecticides in insects, and that many involve point mutations of target genes for insecticide which increase the copy number of the gene, mRNA level and the diversification of the coding sequence^27^. In a study of neonicotinoids, total enzyme activities of the candidate metabolic enzymes were measured in *N. lugens*, indicating that the increase of neonicotinoids resistance was due mainly to P450 monooxygenase, instead of mutations of the nicotinic acetylcholine receptors that were considered as possible targets^28^. Puinean used piperonyl butoxide, an inhibitor specific to P450s, to decrease the level of imidacloprid resistance significantly, which confirmed the important roles of P450s in insecticide resistance^29^. Similarly, the phenomenon was also reported in aphids^30^. Additionally, specific P450 genes have been related to neonicotinoids resistance. For instance, *Drosophila CYP6G1* has been proven to confer imidacloprid resistance^14^,^31^, and Puinean suggested that *CYP6Y3* can confer neonicotinoids resistance to *Myzus persicae*^32^.

The P450 CYP6ER1 has been associated with imidacloprid resistance in *N. lugens*^19^. Here, we found that the mRNA level of *CYP6ER1* was significantly higher in the field-caught resistant strains than in the susceptible strains, which generally coincides with previous references. Susceptibility to imidacloprid increased by 75.61% after RNAi treatment in the experimental population (Fig. 3). Similarly, it has been reported that susceptibility to Deltamethrin in *Locusta migratoria* increased by 92.14% and 72.93% after RNAi knockdown of *CYP409A1* and *CYP409B1*, respectively. Additionally, transgenic expression of *CYP6ER1* in *D. melanogaster* lead to a significant increase of imidacloprid resistance. We therefore conclude that the expression of *CYP6ER1* is sufficient to confer imidacloprid resistance. Of particular note is that fact that, though the expression of *CYP6ER1* in the resistant strain can be induced by imidacloprid treatment, the over-expression of this gene was limited when BPH were treated by a relatively higher dose of imidacloprid. This indicates that the imidacloprid resistance phenotype may be more complex than just the over-expression of a single gene. Thus, it is necessary to review and optimize the traditional strategy of pest control.

P450 monooxygenase is well known for metabolizing xenobiotics, such as insecticides, to less toxic metabolites^33^,^34^. For example, CYP6A1, 6A2, 6A5, and 12A1 all have some manner of cyclodiene epoxidase biochemical activity. Graham and Peterson observed the general topology and tridimensional fold of all cytochromes P450^35^. Only a few strictly conserved residues, as components of a four-helix (D, E, I and L) bundle, two sets of β-sheets, and a coil, participate to form the center of P450s together with a central heme-group bound to the thiolate of a relatively highly conserved cysteine residue, which give access to the biotransfer of electrons and protons to activate oxygen. On the other hand, the individual substrate specificity of the enzymes lies on the individual residues despite their homologous and similar regions, conferring their unique function in reactions with different insecticides.

Over-expression of *CYP6ER1* was able to confer imidacloprid resistance, and this could be induced by certain doses of imidacloprid itself. Therefore, it is important to explore further the interaction between CYP6ER1 and imidacloprid. Utilizing homology modeling technology, and based on the high degree of homology with CYP3A4 in human, we predicted and visualized the molecular interaction wherein nitrogen atoms in the heterocycle of the imidachloprid molecule bond mainly to iron atoms in the center of the homology model via 4, 5-dihedro-1H-imidazole. CYP6CMAvQ and CYP6G1, two identified P450s which detoxify imidachloprid, were referred to our alignment with CYP6ER1 and CYP3A4^26^,^36^, confirming that target sequences and the substrate recognition site (SRS) regions had a high level of similarity^26^. Alignment of analogous imidacloprid binding residues in these four imidacloprid metabolizing enzymes indicated a number of consistent binding residues among them. To assess the stability and the balance of the CYP6ER1-imidacloprid complex, several parameters in the field of energetic and geometric criteria were calculated and analyzed until we obtained an optimized model to simulate the interaction. After optimization, our homology model proved to be of high quality. Additionally, there were some putative key residues responsible for imidacloprid binding with similarity among the four enzymes, most of which create the hydrophobic interface of the binding cavity, as suggested by the docking analysis. Phe-124 and Asp-290 anchor imidacloprid by aromatic interactions, and create a significant part of the contact surface area between imidacloprid and CYP6ER1. Pro-375 (SRS-5) and Pro-289 (SRS-2) likely generate hydrogen bonds to further stabilize imidacloprid. In these residues interacting with imidacloprid, Ala-313 and Gly-314 are conserved in three other imidacloprid metabolizing enzymes: CYP6CM1, CYP3A4 and CYP6G1. The conservation of these residues and the relative large hydrophobic contact surface area, show that residues play an important role in contributing to these enzymes function of metabolizing imidacloprid. However, we cannot ignore the possibility of other residues constructing the model, despite the lack of apparent homologues among these enzymes, which may develop its own specific function and structure for the enzyme.

Our findings have demonstrated the importance of homology modelling and docking to search the potential catalytic center of an enzyme with its substrate. Our research can also lead a strong foundation for future insecticide design. However, a deeper understanding of the interaction mode of imidacloprid related heterocyclic compounds with P450s is needed in order to guide the rational development of insecticide-related compounds and increasing the accuracy and efficiency of computational based drug construction. In addition, the predicted residues and sites involved in detoxifying imidacloprid require further biochemical validation.

## Material and Methods

### Insect strains

The field-caught strains were collected from Nanning, Guangxi, China in 2012, and provided by Institute of Plant Protection, Guangxi Academy of Agricultural Sciences. The susceptible strains were obtained from Zhejiang Academy of Agricultural Sciences in 2012 and reared on rice plants without insecticide treatment. The resistance ratios for imidacloprid of the field-caught strain (LC_50_ 249.13 mg/L) to the susceptible strain (LC_50_ 0.22 mg/L) was 1132-fold.

### Insecticide exposure

Imidacloprid (96.04%) was purchased from Tianyuan Biochemical Co. Ltd (Guangxi, China). We use the rice stem dipping method for insecticide exposure to BPH as described^37^. Different doses of imidacloprid were diluted with acetone to a series of concentrations, and then 1% Tween 80 was added. Rice stems rooted in culture cups were dipped in insecticide solutions for 30 s. Once the stems were dry, one-day-old long-winged female adults were put into each culture cup and fed on these treated rice. Each treatment was performed in triplicate.

### Total RNA extraction

Total RNA was extracted from BPH by using a Total RNA extract kit (Omega). Genomic DNA was digested with DNase I (Takara Bio Inc., Kyoto, Japan). The quality and concentration of RNA samples were examined by agarose gel electrophoresis and spectrophotometric analysis. cDNA was synthesized by reverse transcription in 20 μl reaction volumes containing 1 μg of total RNA by using PrimeScript reverse transcriptase (Takara). The reaction mixture was stored at −20 °C for future use^37^.

### qRT-PCR

Primers were designed for qRT-PCR based on the complete coding sequences of *CYP6ER1* genes as archived in the NCBI database (Table S1). The primers had annealing temperatures of approximately 58 °C. Primers corresponding to the housekeeping gene *β-Actin* were used as endogenous controls^38^. qRT-PCR was performed in a LightCycler480 (Roche, Indianapolis, IN, USA) using a SYBR Premix Ex Taq Kit (Roche) with the following conditions: 95 °C for 5 min, 40 cycles of 95 °C for 10 s, 58 °C for 20 s and 72 °C for 20 s. For the final melting curve step, the samples were subjected to 95 °C for 5 s, held at 65 °C for 1 min and then ramped to 97 °C in 5 °C increments every 1.5 s. A final cooling step held the samples at 40 °C. All qRT-PCR analysis was based on three independent experiments. After three repeat PCR experiments, the gene expression level was calculated using the 2^−△△^CT method^39^.

### RNA interference and bioassays

To synthesize dsRNA, a 529 bp fragment of *CYP6ER1* and a 688 bp fragment of *GFP* [GenBank: ACY56286] were amplified via PCR using the *N.lugens* cDNA as a template. PCR primers are listed in Table S1. The dsRNA synthesis step was performed as described previously^37^. For the RNAi efficiency evaluation, 0.05, 0.1 and 0.2 micrograms of ds*CYP6ER1* were injected into the side of the abdomen of synchronous one-day-old female *N.lugens* macropterous using a 10 μl micro-syringe (Hamilton). The control injection was performed with an equivalent volume of ds*GFP*. Each experimental group had 30 individuals. Ten adults were selected randomly at 24 h, 36 h and 48 h after injection for the independent detection of *CYP6ER1*. Total RNA extraction and qRT-PCR detection were performed as described above. For the susceptibility detection after RNAi, thirty adults from each group were chosen 24 h after injection, with 0.05 micrograms of ddH_2_O, ds*GFP* and ds*CYP6ER1* for each one day-old macropterous female adult. Thirty adults were placed in rice soaked in a solution of 240 ng/μl imidacloprid for 30 seconds. The *N.lugens* mortality was calculated after 96 hours. Each experiment was repeated in triplicate.

### Developmental expression and tissue distribution analysis

The expression of *CYP6ER1* were estimated by qRT-PCR as described^38^ from the first instar nymphs to 13-day-old female adults from the field-caught strain without imidacloprid. To investigate the expression of *CYP6ER1* in different tissues, we extracted the total RNA from the fat body, ovary, midgut, muscle, Malpighian tubule and cuticle. The following steps were performed as described^38^.

### Construction of Transgenic *Drosophila* and Bioassays

The *CYP6ER1* CDS cloned was inserted into a pValium20 vector to prepare UAS-*CYP6ER1* constructs. Subsequently, the constructs were microinjected into the embryos of the y sc v nanos-integrase; attP2 *D. melanogaster* germline using standard techniques in the Center of Biomedical Analysis, Tsinghua University. The transformed line was crossed with anAct5C-GAL4 line for expression of the *CYP6ER1* gene. The genotype of the cross was Act5C >*CYP6ER1*. For use as a control, the transformed line and the Act5C-GAL4 line were crossed with the W^1118^ line, the progenies of which did not express *CYP6ER1* gene with the genotype of UAS- *CYP6ER1* and Act5C-GAL4. RT-PCR was used to confirm the expression of the *CYP6ER1* gene in transgenic *Drosophila* using primers specific for the *CYP6ER1* gene and the reference housekeeping gene *DmActin*^40^. For each cross, total RNA was extracted from 10 adults using the methods described above and three biological replicates were performed for each experiment. The reaction conditions were 94 °C for 5 min, 30 cycles of 94 °C for 30 s, 58 °C for 30 s, and 72 °C for 30 s, using GoTaq G2 Green Master Mix (Promega). Twenty 1–3 day old adults (10 females and 10 males) were used in the contact bioassays, as described previously^41^. In brief, flies were placed in vials with 10 ml corn meal medium containing 300 μg imidacloprid. Three replicates were performed for each assay. After 72 h, the survival rates were calculated and analyzed using the Chi-squared test.

### Homology modelling

The structure of CYP6ER1 was predicted using the Homology Modeling protocol of Discovery Studio 2.5. A BLAST search against the PDB_nr95 database was used to search the homology sequence by using the E-value 10–10 as a parameter. A multi sequence alignment between CYP6ER1 and homology sequences was performed to obtain the best reference for the homology model building. An ideal homologous model should cover as much of the Alignment Length of our target sequence as possible, while keeping relatively high Positive and Sequence identity, which was also analyzed via Align Multiple Sequence in Discovery Studio with default parameters. The crystal structure of human CYP3A4 with the highest resolution and good stereochemistry was used to build the homology model. The best model for subsequent optimization was chosen according to the total PDF energy, PDF physical energy and DOPE score, using Cut overhangs, refine loops and the use DOPE method as parameters.

### Molecular dynamics simulations

According to the covalent bonds between ritonavir (RIT) and CYP3A4, we speculated that bonding also occurred between imidacloprid (IM4) and CYP6ER1, though conventional docking programs cannot be utilized to generate the ligand-bound conformations. Observing charge distribution in local structures of imidacloprid and CYP6ER1 calculated by the Gaussian-09 program at the HF/6-31G* level (Gaussian 09, revision a.02. Gaussian Inc., Wallingford)^42^, we found 4, 5-dihedro-1H-imidazole in structural imidacloprid was partly similar to the thiazole structures of RIT. Therefore, it is possible that nitrogen atoms in the heterocycle of the imidachloprid molecule bonded to iron atoms via 4, 5-dihedro-1H-imidazole. A comparison of the electrostatic distributions of RIT and IM4 suggests that IM4 likely has the same binding mode as RIT, and IM4 was found to be manually docked into CYPYER1. A molecular dynamics (MD)-based optimization assessment of the complex structure of CYPYER1 protein was performed with the Discovery Studio 2.5 Dynamics (Heating or Cooling, Equilibration and Production) Module under the CHARMm force field^43^. The partial charge and implicit solvent model were set to Momany-Rone and generalized born as the molecular dynamics (MD)-based optimization parameters, Electrostatics as Spherical cutoff, Apply SHAKE constraint as True, Minimization as 400, Heating as 2,000, Equilibration as 2,000, Production as 100,000 and other parameters as the defaults.

## Additional Information

**How to cite this article**: Pang, R. *et al*. Functional analysis of *CYP6ER1*, a P450 gene associated with imidacloprid resistance in *Nilaparvata lugens*. *Sci. Rep*. **6**, 34992; doi: 10.1038/srep34992 (2016).

## Supplementary Material

Supplementary Information

## Figures and Tables

**Table 1:** Alignment of the analogous imidacloprid binding residues in four imidacloprid metabolizing enzymes: CYP6ER1, CYP6CM1, CYP3A4, CYP6G1.

*CYP6ER1*	*CYP6CM1*	*CYP3A4*	*CYP6G1*	SRS location	Secondary structure
124Phe	130Phe	98Ser	124Phe	SRS-1	Loop between helices B and C
288Asp	224PHe	190Leu	218Phe	SRS-2	Loop between helices F and F’
289Pro	225Arg	191Arg	219Thr	SRS-2	Loop between helices F and F’
290Asp	226Phe	192Phe	220Phe	SRS-2	Loop between helices F and F’
246Phe	251Ala	219Val	245Phe	SRS-3	Loop between helices G’ and G
247Thr	252Val	220Phe	246Phe	SRS-3	Loop between helices G’ and G
312Leu	321Ser	283Phe	311Thr	SRS-4	Helix I (O_2_ binding motif)
313Ala	322Ala	284Ala	312Ala	SRS-4	Helix I (O_2_ binding motif)
314Gly	323Gly	285Gly	313Gly	SRS-4	Helix I (O_2_ binding motif)
317Thr	326Pro	288Thr	316Thr	SRS-4	Helix I (O_2_ binding motif)
374Gly	387Ala	348ILe	377Val	SRS-5	Loop between helix K and b-strand
375Pro	388Ser	349Ala	378Leu	SRS-5	Loop between helix K and b-strand
376Val	389Gly	350Met	379Pro	SRS-5	Loop between helix K and b-strand
487Thr	501Ser	460Gly	497Gly	SRS-6	C-terminal loop
488Phe	502Phe	461Gly	498Phe	SRS-6	C-terminal loop
489Val	503Thr	462Leu	499Val	SRS-6	C-terminal loop

Analyses were based on structure sequence alignment between the CYP6CM1 model and the CYP3A4 crystallographic structure together with sequence alignment to CYP6G1. Some of the residues that contribute to the stabilization of imidacloprid in the active site are also important in the stabilization of the heme and oxygen molecules.

## References

[b1] Matsumura M. & Sanada-Morimura S. Recent Status of Insecticide Resistance in Asian Rice Planthoppers. Jarq-Jpn Agr Res Q 44, 225–230 (2010).

[b2] Georghiou G. P. & Mellon R. B. In Pest Resistance to Pesticides. (eds. G.P. Georghiou & T. Saito) 1–46 (Springer: US, Boston, MA, ; 1983).

[b3] Nagata T. Monitoring on Insecticide Resistance of the Brown Planthopper and the White Backed Planthopper in Asia. Journal of Asia-Pacific Entomology 5, 103–111 (2002).

[b4] Davies T. G., Field L. M., Usherwood P. N. & Williamson M. S. DDT, pyrethrins, pyrethroids and insect sodium channels. IUBMB Life 59, 151–162 (2007). 17487686 10.1080/15216540701352042

[b5] Kimura Y. Resistance to Malathion in the Small Brown Planthopper, *Laodelphax striatellus FALLÉN*. Japanese Journal of Applied Entomology & Zoology 9, 251–258 (1965).

[b6] Zibaee A., Sendi J. J., Ghadamyari M., Alinia F. & Etebari K. Diazinon resistance in different selected strains of *Chilo suppressalis* (Lepidoptera: Crambidae) in northern Iran. J Econ Entomol 102, 1189–1196 (2009). 19610437 10.1603/029.102.0343

[b7] Dai S. M. & Sun C. N. Pyrethroid Resistance and Synergism in *Nilaparvata lugens Stål* (Homoptera: Delphacidae) in Taiwan. Journal of Economic Entomology 77, 891–897 (1984).

[b8] Su J. Y. . Status of Insecticide Resistance of the Whitebacked Planthopper, *Sogatella Furcifera* (Hemiptera: Delphacidae). Fla Entomol 96, 948–956 (2013).

[b9] Gorman K., Liu Z., Denholm I., Bruggen K. U. & Nauen R. Neonicotinoid resistance in rice brown planthopper, Nilaparvata lugens. Pest Management Science 64, 1122–1125 (2008). 18803175 10.1002/ps.1635

[b10] Zhao X. . Differential resistance and cross-resistance to three phenylpyrazole insecticides in the planthopper *Nilaparvata lugens* (Hemiptera: Delphacidae). J Econ Entomol 104, 1364–1368 (2011). 21882705 10.1603/EC11074

[b11] Gorman K. . Cross-resistance relationships between neonicotinoids and pymetrozine in *Bemisia tabaci* (Hemiptera: Aleyrodidae). Pest Management Science 66, 1186–1190 (2010). 20632380 10.1002/ps.1989

[b12] Feyereisen R. Insect P450 enzymes. Annu Rev Entomol 44, 507–533 (1999). 9990722 10.1146/annurev.ento.44.1.507

[b13] Farnham A. W. The Mode of Action of Piperonyl Butoxide with Reference to Studying Pesticide Resistance - Piperonyl Butoxide - 12. Piperonyl Butoxide, 199–213 (1999).

[b14] Daborn P. J. . A single p450 allele associated with insecticide resistance in *Drosophila*. Science 297, 2253–2256 (2002). 12351787 10.1126/science.1074170

[b15] Liu N. & Scott J. G. Increased transcription of CYP6D1 causes cytochrome P450-mediated insecticide resistance in house fly. Insect Biochem Mol Biol 28, 531–535 (1998). 9753764 10.1016/s0965-1748(98)00039-3

[b16] Terhzaz S. . A novel role of Drosophila cytochrome P450-4e3 in permethrin insecticide tolerance. Insect Biochem Mol Biol 67, 38–46 (2015). 26073628 10.1016/j.ibmb.2015.06.002 PMC4673087

[b17] Crossthwaite A. J., Rendine S., Stenta M. & Slater R. Target-site resistance to neonicotinoids. J Chem Biol 7, 125–128 (2014). 25320645 10.1007/s12154-014-0116-y PMC4182339

[b18] Liu Z. W. . Selection for imidacloprid resistance in *Nilaparvata lugens*: cross-resistance patterns and possible mechanisms. Pest Management Science 59, 1355–1359 (2003). 14667058 10.1002/ps.768

[b19] Bass C. . Overexpression of a cytochrome P450 monooxygenase, CYP6ER1, is associated with resistance to imidacloprid in the brown planthopper, Nilaparvata lugens. Insect Mol Biol 20, 763–773 (2011). 21929695 10.1111/j.1365-2583.2011.01105.x

[b20] Bao H. B. . The roles of CYP6AY1 and CYP6ER1 in imidacloprid resistance in the brown planthopper: Expression levels and detoxification efficiency. Pestic Biochem Phys 129, 70–74 (2016). 10.1016/j.pestbp.2015.10.020 27017884

[b21] Chiu T. L., Wen Z., Rupasinghe S. G. & Schuler M. A. Comparative molecular modeling of *Anopheles gambiae* CYP6Z1, a mosquito P450 capable of metabolizing DDT. Proc Natl Acad Sci USA 105, 8855–8860 (2008). 18577597 10.1073/pnas.0709249105 PMC2449330

[b22] Lertkiatmongkol P., Jenwitheesuk E. & Rongnoparut P. Homology modeling of mosquito cytochrome P450 enzymes involved in pyrethroid metabolism: insights into differences in substrate selectivity. BMC Research Notes 4, 1–7 (2011). 21892968 10.1186/1756-0500-4-321 PMC3228512

[b23] Baudry J., Rupasinghe S. & Schuler M. A. Class-dependent sequence alignment strategy improves the structural and functional modeling of P450s. Protein Eng Des Sel 19, 345–353 (2006). 16777908 10.1093/protein/gzl012

[b24] de Graaf C., Vermeulen N. P. E. & Feenstra K. A. Cytochrome P450 in silico: An integrative modeling approach. J Med Chem 48, 2725–2755 (2005). 15828810 10.1021/jm040180d

[b25] Jones R. T. . Homology modelling of *Drosophila* cytochrome P450 enzymes associated with insecticide resistance. Pest Management Science 66, 1106–1115 (2010). 20583201 10.1002/ps.1986

[b26] Karunker I. . Structural model and functional characterization of the *Bemisia tabaci* CYP6CM1vQ, a cytochrome P450 associated with high levels of imidacloprid resistance. Insect Biochem Molec 39, 697–706 (2009). 10.1016/j.ibmb.2009.08.006 19716416

[b27] Li X., Schuler M. A. & Berenbaum M. R. Molecular mechanisms of metabolic resistance to synthetic and natural xenobiotics. Annu Rev Entomol 52, 231–253 (2007). 16925478 10.1146/annurev.ento.51.110104.151104

[b28] Rauch N. & Nauen R. Identification of biochemical markers linked to neonicotinoid cross resistance in *Bemisia tabaci* (Hemiptera : Aleyrodidae). *Arch* Insect Biochem 54, 165–176 (2003). 10.1002/arch.10114 14635178

[b29] Puinean A. M., Denholm I., Millar N. S., Nauen R. & Williamson M. S. Characterisation of imidacloprid resistance mechanisms in the brown planthopper, Nilaparvata lugens Stal (Hemiptera: Delphacidae). Pestic Biochem Phys 97, 129–132 (2010).

[b30] Philippou D., Field L. & Moores G. Metabolic enzyme(s) confer imidacloprid resistance in a clone of *Myzus persicae* (Sulzer) (Hemiptera: Aphididae) from Greece. Pest Management Science 66, 390–395 (2010). 19950404 10.1002/ps.1888

[b31] Le Goff G. . Xenobiotic response in *Drosophila melanogaster*: sex dependence of P450 and GST gene induction. Insect Biochem Mol Biol 36, 674–682 (2006). 16876710 10.1016/j.ibmb.2006.05.009

[b32] Puinean A. M. . Amplification of a Cytochrome P450 Gene Is Associated with Resistance to Neonicotinoid Insecticides in the Aphid *Myzus persicae*. Plos Genet 6 (2010). 10.1371/journal.pgen.1000999 PMC2891718 20585623

[b33] Lesca P., Rafidinarivo E., Lecointe P. & Mansuy D. A class of strong inhibitors of microsomal monooxygenases: The elupticines. Chemico-Biological Interactions 24, 189–197 (1979). 428009 10.1016/0009-2797(79)90007-3

[b34] Hedley D., Khambay B. P. S., Hooper A. M., Thomas R. D. & Devonshire A. L. Proinsecticides effective against insecticide-resistant peach-potato aphid (*Myzus persicae* (*Sulzer*)). Pesticide Science 53, 201–208 (1998).

[b35] Graham S. E. & Peterson J. A. How similar are P450s and what can their differences teach us? Arch Biochem Biophys 369, 24–29 (1999). 10462437 10.1006/abbi.1999.1350

[b36] Joussen N., Heckel D. G., Haas M., Schuphan I. & Schmidt B. Metabolism of imidacloprid and DDT by P450 GYP6G1 expressed in cell cultures of *Nicotiana tabacum* suggests detoxification of these insecticides in CYP6G1-overexpressing strains of *Drosophila melanogaster*, leading to resistance. Pest Management Science 64, 65–73 (2008). 17912692 10.1002/ps.1472

[b37] Pang R. . Identification of promoter polymorphisms in the cytochrome P450 CYP6AY1 linked with insecticide resistance in the brown planthopper, Nilaparvata lugens. Insect Mol Biol 23, 768–778 (2014). 25124988 10.1111/imb.12121

[b38] Chen J. . Feeding-based RNA interference of a trehalose phosphate synthase gene in the brown planthopper, Nilaparvata lugens. Insect Mol Biol 19, 777–786 (2010). 20726907 10.1111/j.1365-2583.2010.01038.x

[b39] Livak K. J. & Schmittgen T. D. Analysis of relative gene expression data using real-time quantitative PCR and the 2(T)(-Delta Delta C) method. Methods 25, 402–408 (2001). 11846609 10.1006/meth.2001.1262

[b40] Ponton F., Chapuis M. P., Pernice M., Sword G. A. & Simpson S. J. Evaluation of potential reference genes for reverse transcription-qPCR studies of physiological responses in *Drosophila melanogaster*. J Insect Physiol 57, 840–850 (2011). 21435341 10.1016/j.jinsphys.2011.03.014

[b41] Daborn P., Boundy S., Yen J., Pittendrigh B. & ffrench-Constant R. DDT resistance in *Drosophila* correlates with Cyp6g1 over-expression and confers cross-resistance to the neonicotinoid imidacloprid. Mol Genet Genomics 266, 556–563 (2001). 11810226 10.1007/s004380100531

[b42] Caricato M., Trucks G. W. & Frisch M. J. On the difference between the transition properties calculated with linear response- and equation of motion-CCSD approaches. J Chem Phys 131 (2009). 10.1063/1.3255990 19894995

[b43] Brooks B. R. . CHARMM: The Biomolecular Simulation Program. J Comput Chem 30, 1545–1614 (2009). 19444816 10.1002/jcc.21287 PMC2810661

