# Supplementary material for: Functional analysis of CYP6ER1, a P450 gene associated with imidacloprid resistance in Nilaparvata lugens
Source: Sci Rep. 2016 Oct 10;6:34992. doi: 10.1038/srep34992 (PMC5056347; doi:10.1038/srep34992)
Supplement: Supplementary Information [file srep34992-s1.pdf]

Supplementary Information

Functional analysis of *CYP6ER1*, a P450 gene associated with imidacloprid resistance in *Nilaparvata lugens*

Author information

Rui Pang[a1], Meng Chen[a1], Zhikun Liang[a], Xiangzhao Yue[a], Hu Ge[b], Wenqing Zhang[a]*.

a. State Key Laboratory of Biocontrol, School of Life Sciences, Sun Yat-sen University, Guangzhou, China, 510006.

b. School of Pharmaceutical Science, Sun Yat-sen University, Guangzhou, China, 510006

* Corresponding author: Wenqing Zhang

E-mail: lsszwq@mail.sysu.edu.cn

Tel: (+86) 020 39332963,

Fax: (+86) 020 39943515.

1 Both authors contributed equally to this work and should be considered co-first authors

E-mail addresses:

Rui Pang: pr839@163.com

Meng Chen: mytinco@qq.com

Zhikun Liang: pardonliang@126.com

Xiangzhao Yue: kathy126@yeah.net

Hu Ge: gehuchina@gmail.com

Table S1. PCR primers used in this study.

Primers name	Purpose	Primer sequence
6ER1-RNAi-F	ds*CYP6ER1* synthesis	5'-CCCCCTCTCACACCATCTGTT-3′
6ER1-RNAi-R	ds*CYP6ER1* synthesis	5'-CGACTCTTTGCTTTTTTCAATCTCC-3′
GFP-RNAi-F	ds*GFP* synthesis	5'-AAGGGCGAGGAGCTGTTCACCG-3′
GFP-RNAi-R	ds*GFP* synthesis	5'-CAGCAGGACCATGTGATCGCGC-3′
ACTIN-F	RT-PCR (Actin)	5'-TGCGTGACATCAAGGAGAAGC-3′
ACTIN-R	RT-PCR (Actin)	5'- CCATACCCAAGAAGGAAGGCT-3′
6ER1-dsRNA-RT-F	RT-PCR (*CYP6ER1* in RNAi research)	5'-ATTCCGGTCTATGCGCTTC-3′
6ER1-dsRNA-RT-R	RT-PCR (*CYP6ER1* in RNAi research)	5'-TGGATTGGCGCTCTCTTACT-3′
6ER1-RT-F	RT-PCR (*CYP6ER1*, which was cloned in this research)	5'-TTCCGTTAGTGCGTTGGTTAC-3′
6ER1-RT-R	RT-PCR (*CYP6ER1,* which was cloned in this research)	5'-AATGGTTCTCCCACTGGATTT-3′

Table S2. BLAST search and sequence alignment.

Protocol		PSI-BLAST Search
Parameters	Target sequence	Cytochrome P450 *CYP6ER1*
	Database	PDB_nr95
	E-value Cutoff	10
	Maximum Hits	100
	Scoring Matrix	BLOSUM62
Result	PDB code	3NXU
	Identity	31%
	Sequence length	457
	Alignment length	480
	E-value	1.63273e-91
	Positive	52
	Resolution	2
	SCOP	a.104.1.1
	Ligand	HEM and RIT
	Description	Cytochrome P450 3A4

Table S3. Model scores from the homology modeling of *CYP6ER1.*

Name	PDF Total Energy	PDF Physical Energy	DOPE Score
6ER1.M0011	4880.4561	1866.3110218	-52366.699219
6ER1.M0016	4991.5415	1956.281103	-52570.093750
6ER1.M0003	5013.8979	1924.61454914	-51440.234375
6ER1.M0009	5061.2505	2031.34542017241	-51891.925781
6ER1.M0014	5069.5327	1947.6158598	-52380.808594
6ER1.M0008	5095.7954	1914.9310658	-51684.746094
6ER1.M0005	5098.7485	1948.59656096	-51379.964844
6ER1.M0017	5154.1743	2079.17296940001	-51415.792969
6ER1.M0015	5168.4302	2012.83729205	-51804.324219
6ER1.M0001	5178.3896	1975.23588675	-51668.664063
6ER1.M0018	5230.6372	1987.3336503	-51077.031250
6ER1.M0007	5249.3813	2101.75486200001	-52651.351563
6ER1.M0019	5286.7007	2144.36547100024	-50818.742188
6ER1.M0006	5374.6938	2185.4297581	-51437.210938
6ER1.M0012	5381.5293	2126.134128	-51458.792969
6ER1.M0020	5406.6074	2088.49494070001	-51285.609375
6ER1.M0010	5543.0605	2130.575033245	-50681.925781
6ER1.M0002	5580.2217	2235.2910817	-51170.558594
6ER1.M0004	5736.0063	2166.807654	-50950.031250
6ER1.M0013	5754.1113	2080.516687	-51081.066406

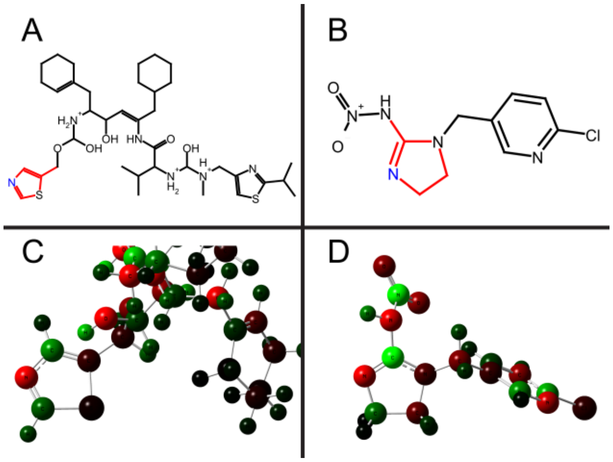

Figure S1. Molecular structure and approximate comparison of the ESP charge distribution of RIT (A, C) and IM4 (B, D).

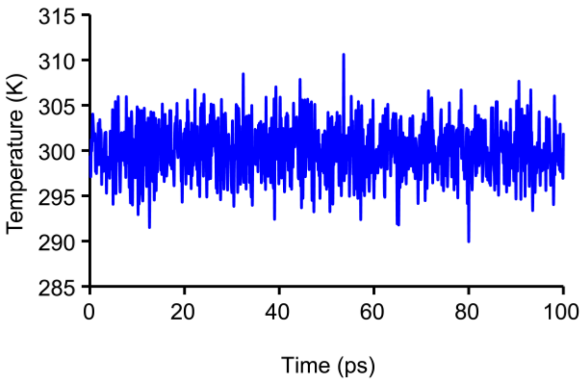

Figure S2. Temperature (K) fluctuation versus time (ps) during the 100 ps of MD simulation production

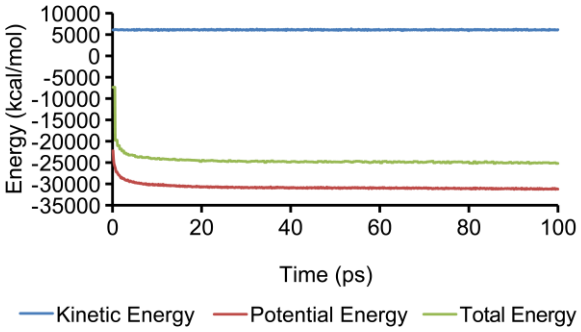

Figure S3. The total energy, kinetic energy and Molential energy (kcal/mol) fluctuations versus time (ps).

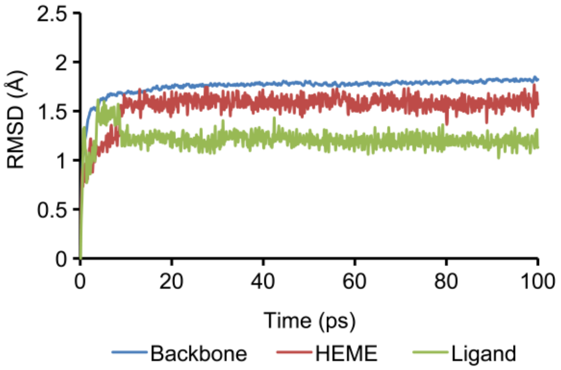

Figure S4. RMSD of atomic coordination of protein backbone, HEME and ligand.
